# An Investigation of the Contact Fatigue Characteristics of an RV Reducer Crankshaft, Considering the Hardness Gradients and Initial Residual Stress

**DOI:** 10.3390/ma15217850

**Published:** 2022-11-07

**Authors:** Xin Li, Wen Shao, Jinyuan Tang, Han Ding, Weihua Zhou

**Affiliations:** State Key Laboratory of High Performance Complex Manufacturing, College of Mechanical and Electrical Engineering, Central South University, Changsha 410083, China

**Keywords:** RV reducer crankshaft, initial residual stress, hardness gradients, rolling contact fatigue, modified Fatemi–Socie criterion

## Abstract

The crankshaft is one of the core components of a Rotate Vector (RV) reducer. The fatigue life of the RV reducer is severely hindered by fatigue failure on the eccentric cylindrical surface of the crankshaft. The hardness gradients and residual stress in the crankshaft, associated with machining operations, exert an enormous impact on the rolling contact fatigue (RCF). In this work, a finite element method (FEM)-based three-dimensional elasto-plastic contact model is established to calculate the stress–strain field by taking hardness gradients and initial residual stress into account. The RCF characteristics of an RV reducer crankshaft is investigated by applying modified Fatemi–Socie (FS) multiaxial fatigue criterion. The results indicate that initial residual stress plays an influential role in the fatigue damage by altering the distribution of the maximum normal stress near the contact surface. The modified FS fatigue criterion could better consider the effect of initial residual stress and the shear stress, which significantly improves the prediction accuracy of the contact fatigue life model. The contact fatigue performance could be considerably improved by designing appropriate shot peening parameters to obtain optimized residual stress distribution. Therefore, the technique presented may serve as an important guideline for the anti-fatigue design of an RV reducer crankshaft.

## 1. Introduction

The RV (Rotate Vector) reducer is extensively used in industry robots owing to its peculiar and fascinating properties, such as compact structure, small size, light weight, high reduction ratio, high transmission accuracy and efficiency, high torsional rigidity, etc. [1]. The crankshaft and cylindrical roller bearing are the core components of the RV reducer. Due to the limited available space of the RV reducer, the cylindrical roller bearing is usually without the inner ring and outer ring. That is, the inner and outer rings of the bearing are directly composed of the eccentric cylindrical surface of the crankshaft and the bearing inner hole on the cycloid wheel. The transmission errors and fatigue life of the RV reducer is severely hindered by failure modes, such as pitting and spalling on the eccentric cylindrical surface caused by the long-term high cyclic contact stress between the eccentric cylindrical surface of the crankshaft and the roller bearing [2]. With increasingly higher performance requirements, such as high transmission accuracy, high load-carrying capacity, and long service life of the RV reducer, the RCF of the crankshaft has also become the limiting factor affecting the reliability of the RV reducer.

Several attempts have been made towards improving the transmission performance of RV reducers. Zhang et al. [3] established a mixed lubrication analysis model for RV reducers. The contact load, surface roughness, and geometry of the cylindrical roller bearings were innovatively included in their model. Xu et al. [4,5] developed a dynamic model for the transmission systems of an RV reducer that took into account the cylindrical roller bearing’s radial clearance. Wang et al. [6] presented a contact force and transmission error analysis of an RV Reducer. Their results revealed that the modified model, based on contact force curves, could improve the transmission performance of the RV reducer as the load increased. Deng et al. [7] calculated the rated life of the RV reducer’s angular contact ball bearing. They also discovered that bearing life had a significant impact on the total life of the RV reducer under heavy load conditions. The present investigations on RV reducers have been mostly focused on the meshing characteristics of the cycloid pinwheel, the dynamic characteristics of the RV reducer, and the life estimation of the bearing. Few studies have been reported on the RCF performances of the crankshaft of the RV reducer due to the complexity of the contact load between the RV reducer crankshaft and the cylindrical roller bearing, although it exerts an enormous impact on the reliability and fatigue life of the RV reducer.

Concerted efforts were directed towards the fatigue life and damage prediction methods for gears and rolling bearings. Li et al. [8] presented a multiaxial fatigue model considering mixed lubrication for crack initiation life prediction of spur gears. Liu et al. [9] proposed an improved multiaxial fatigue life model with higher life prediction accuracy compared with classical models. Vijay et al. [10] presented a novel model to simulate the crack initiation and propagation in bearing steels, considering the anisotropy of crystal. Their results indicated that the Fatemi–Socie (FS) criterion could be used to estimate the RCF life. Continuum damage mechanics (CDM) and the elasto-plastic model with damage-coupling were widely used to investigate the spalling initiation and propagation behaviors of cylindrical rolling bearings [11,12]. Despite the efforts of these earlier studies, prediction of the fatigue life and damage from these investigations may inevitably suffer from the disadvantage of neglecting the effect of mechanical property that is introduced in the manufacturing processes, such as heat treatment, grinding, shot peening, etc.

In recent years, considerable progress has been achieved for the evaluation of the contact fatigue performance by taking surface integrity such as surface topography, residual stress, hardness gradient, and microstructure into account. Numerous researchers have shown that surface integrity significantly affects the fatigue life in rolling contact [13]. Choi et al. [14] pointed out that the prediction accuracy of fatigue life increased more than 40% when the residual stress was taken into account. The results also showed that increments of more than 12 and 8 times could be reached for the crack initiation propagation lives if residual stress was considered. The critical plane approach was recognized as an effective method to solve multiaxial fatigue problems [9]. The Dang–Van fatigue criterion, the FS criterion, and Zaretsky fatigue life model were applied to study the contact fatigue performance of a carburized gear under heavy loading by incorporating residual stress [15,16]. Mahdavi et al. [17] investigated the effect of superposed residual stresses on micro plasticity around inclusions in bearing steel. Ooi et al. [18] experimentally studied the impact of restrained austenite and residual stress on the fatigue life of carburized AISI 8620 steel. Although residual stress was considered in [17,18], the distribution of residual stress was not well represented in these studies for the estimation of RCF. Guan et al. [19] investigated the influence of compressive residual stress (CRS) induced by shot peening on fatigue risks and found that appropriate CRS distribution could decrease the rate of damage accumulation in bearing steel containing carbide. Walvekar et al. [20] studied the combined impact of hardness gradient and residual stress curves on the RCF lives of bearing steel materials. It was found that optimized carburizing depth could prolong the fatigue life of bearing steel materials to a large extent. Furthermore, the relationship between associated parameters such as effective case hardening depth (CHD), surface hardness, and hardness curve shape and fatigue performance was also demonstrated [21,22].

The above studies confirm that it is of theoretical value and engineering significance to consider the mechanical property gradient and initial residual stress when evaluating the contact fatigue performance of carburized steel materials or components. The researchers above also provided a theoretical basis for the contact fatigue characteristic analysis of the crankshaft. Moreover, the successful prediction of the fatigue life of the crankshaft incorporating residual stress and hardness gradient through numerical modelling provides the superiorities of yielding numerous results in a short period and sometimes offers beneficial insight into different states of RCF processes, which can be also used to optimize the machining parameters. Therefore, it is of great importance and necessity to consider the initial state of the crankshaft in the contact fatigue life prediction model.

In this work, focusing on fatigue life assessment of an RV reducer crankshaft, an FEM-based three-dimensional elasto-plastic contact model is established. The hardness gradients and initial residual stress are obtained by Vickers hardness tests and X-ray diffraction method. The collected hardness and residual stress data are incorporated into the elasto-plastic contact model and then the stress–strain histories are obtained. Contact fatigue life assessment is performed by the critical plane method and multiaxial fatigue criterion. The influence of friction factor and initial residual stress on fatigue damage and fatigue life is investigated. Moreover, the effect of residual stress induced by shot peening on improving fatigue life is also analyzed quantitatively.

## 2. Failure Analysis of Crankshaft

The reliability and fatigue life are important performance indicators of the RV reducer, so it is necessary to carry out fatigue life tests of the RV reducer. In addition, the fatigue life test is not only conducive to analyzing the performance degradation law and failure mechanism of the reducer, but also provides guidance for the design and manufacture of key parts of the RV reducer.

**Figure 1:**
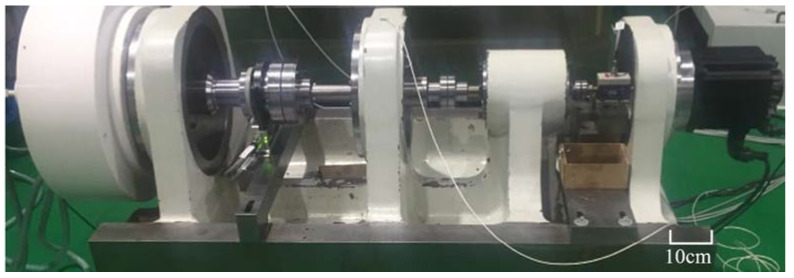
Fatigue life test system of RV Reducer.

Figure 1 shows fatigue life test system of an RV reducer. Because the RV reducer is a piece of high-precision transmission equipment, transmission efficiency can be selected as the failure judgment criterion, and comprehensive judgment can be made in combination with vibration, temperature rise, and noise. One RV reducer testing machine is selected for fatigue test. The test is stopped when the transmission efficiency is detected to be lower than 85% of the threshold, and a service life of approximately 2150 h was obtained.

**Figure 2:**
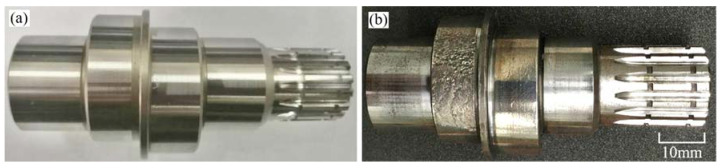
Macroscopic morphology of crankshaft: (**a**) the new part; (**b**) the failed part.

The reducer was disassembled after the fatigue test. Comparing the new crankshaft parts, it was found that the cylindrical surface of the needle roller bearing in contact with the crankshaft was seriously worn, as shown in Figure 2.

**Figure 3:**
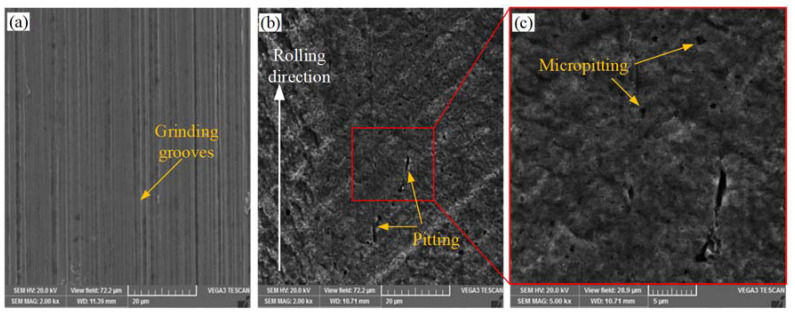
The SEM micrographs of the eccentric cylindrical surface of the crankshaft: (**a**) surface of new parts; (**b**,**c**) surface after failure.

The SEM micrographs of the eccentric cylindrical surface of the crankshaft before and after failure are shown in Figure 3. Regular grinding grooves are left on the new crankshaft surface (as shown in Figure 3a). There are thin strip-like scratches along the rolling direction, and numerous pit-like cracks appeared on the surface of the failed crankshaft (Figure 3b,c).

Because the crank shaft is the core part of the power input and output of the reducer, it bears periodic radial load and dynamic load. Moreover, the reducer often experiences impact load, continuous start and stop, and other working conditions, which leads to complex load changes on the crankshaft. Therefore, the eccentric cylindrical surface of the crankshaft is also the most prone to failure in engineering practice. Fatigue pitting occurs on the eccentric cylindrical surface of the crankshaft under the long-term high cyclic contact stress, and a series of pits are formed. The crankshaft is one of the weakest links of the RV reducer, which severely restricts the fatigue life of the RV reducer.

## 3. Methodology

### 3.1. Stress Analysis of RV Reducer

**Figure 4:**
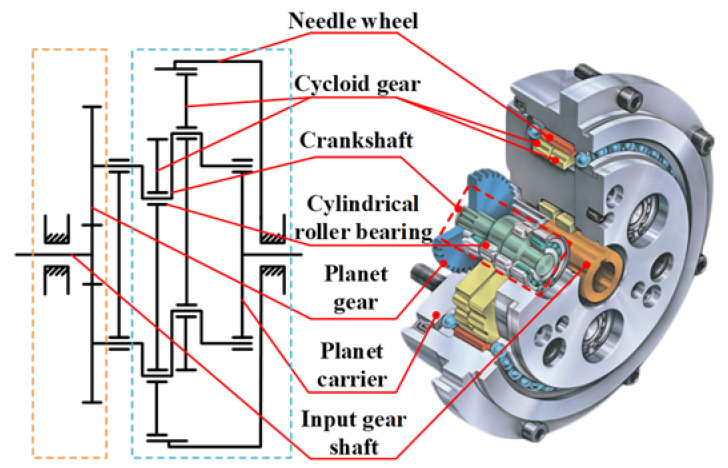
Transmission schematic diagram of RV Reducer.

**Figure 5:**
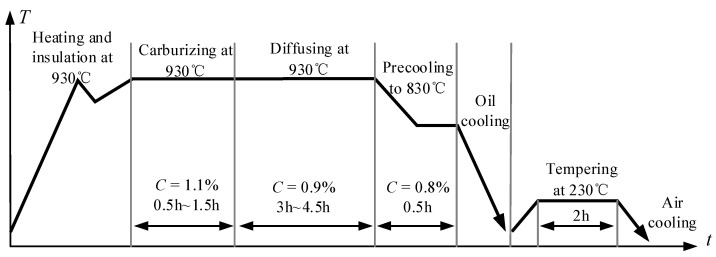
The curve of the thermal treatment process.

The crankshaft used in this study is taken from the RV reducer of an industrial robot. The structure of the RV reducer is shown in Figure 4. The critical parameters of the rolling contact pair between the crankshaft and roller are given in Table 1. The crankshaft material is 20CrNi2MoA. The composition of it is listed in Table 2. The crankshaft has undergone several manufacturing processes, such as carburizing, quenching, tempering, and finally precision grinding. The detailed thermal treatment process is shown in Figure 5. The crankshaft sample is carburized and diffused for 6 h after the temperature soared to 930 °C, then the sample is quickly quenched in oil, followed by low temperature tempering at 230 °C for 2 h. After heat treatment, the lath martensite structure is finally obtained. Lath martensite can better resist impact and crack propagation, so that the material has higher hardness, good wear resistance, and higher contact fatigue properties.

**Figure 6:**
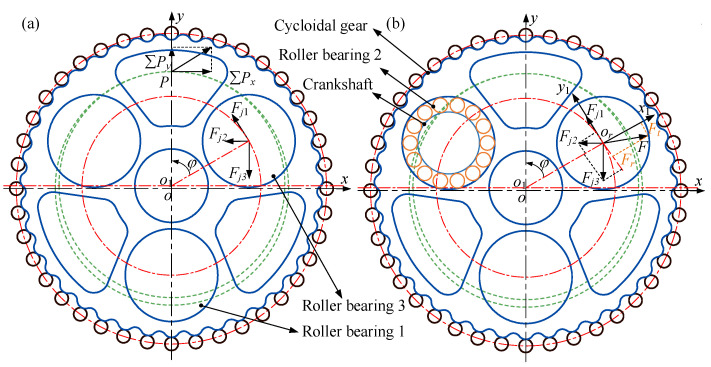
Schematic diagram: (**a**) force on cycloid gear; (**b**) force on cylindrical roller bearing.

Through the motion and force analysis of the cycloidal gear and cylindrical roller bearing (Figure 6), the resultant force acting on the cylindrical roller bearing is obtained. The cylindrical roller bearing bears the force from the cycloidal gear and the crankshaft. *F_j_*_1_, *F_j_*_2_, and *F_j_*_3_ are the components of force, *F*, which acts on the cycloidal gear via the cylindrical roller bearing (as shown in Figure 6a). *F* can also be decomposed into the normal force, *F_r_*, and tangential force, *F_t_* (as shown in Figure 6b).

Based on the structural parameters of the RV reducer and the force equilibrium, *F* can be expressed as follows, and the detailed calculation process can be seen in [23].

(1) $$F=\sqrt{F_{t}^{2}+F_{r}^{2}}=\frac{T_{out}}{2nez_{5}r_{o}}\sqrt{{\left(ez_{5}\right)}^{2}+r_{o}^{2}+k_{y}^{2}r_{o}^{2}+2ez_{5}r_{o}\cos\varphi -2k_{y}ez_{5}r_{o}\sin\varphi }$$

**Figure 7:**
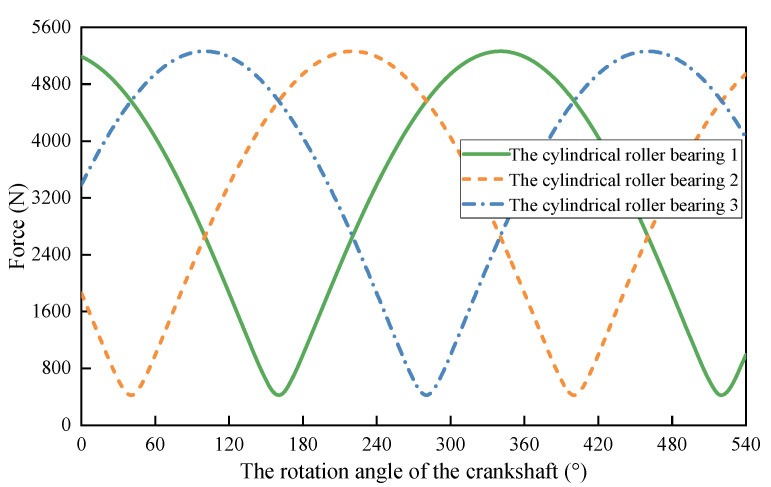
The variation of forces acting on the cylindrical roller bearings.

Combining Table 1 and Equation (1), the resultant force distribution of the cycloid gear acting on the cylindrical roller is obtained, as depicted in Figure 7. Periodic sinusoids are found for the forces acting on the cylindrical rollers. The load fluctuation of three cylindrical roller bearings in the same cycloid gear are found to have a phase difference of 120°. According to Harris et al. [24], the equivalent load of cylindrical roller bearings can be calculated as [23]:

(2) $$F_{m}=\frac{T_{out}}{2}{\left(\frac{{\left(ez_{5}\right)}^{4}+4\left(1+k_{y}^{2}\right){\left(ez_{5}\right)}^{2}r_{o}^{2}+{\left(1+k_{y}^{2}\right)}^{2}r_{o}^{4}}{n^{4}{\left(ez_{5}\right)}^{4}r_{o}^{4}}\right)}^{1/4}$$

Power is transmitted between the crankshaft and the cylindrical rollers. Dynamic loads and relatively large radial forces act on the cylindrical rollers. The force analysis of each roller on the cylindrical roller bearing is carried out after obtaining the resultant force on the cylindrical roller bearing. The Newton–Raphson algorithm is employed to calculate the load distribution of each rolling element acting on the crankshaft. The crankshaft is subjected to periodic load, hence the equivalent contact load acting on the crankshaft by the cylindrical roller bearing will be used as input in the following section.

### 3.2. Modeling of Residual Stress and Hardness Gradient

The *x*, *y*, and *z* directions in the crankshaft coordinates act along the rolling, depth, and axial directions, respectively. Several empirical methods for fitting the hardness distribution profile introduced by carburizing have been proposed by Lang and Kernen [25] and Thomas [26]. Among them, the Thomas method is the closest to the measured results and has been widely used in ISO standards. The empirical formula can be expressed as [26]:

(3) $$HV(y)=\left\{ \begin{array}{l} a_{a}\cdot y^{2}+b_{a}\cdot y+c_{a}(0\leq y<CHD)\\ a_{b}\cdot y^{2}+b_{b}\cdot y+c_{b}(CHD\leq y<y_{cor})\\ HV_{cor}(y_{cor}\leq y) \end{array}\right.$$

(4) $$\left\{ \begin{array}{l} a_{a}=\frac{550-HV_{sur}}{CHD^{2}-2\cdot y_{HV,\max}\cdot CHD};b_{a}=-2\cdot a_{a}\cdot y_{HV,\max};c_{a}=HV_{sur};\\ a_{b}=\frac{H^{\prime }(CHD)}{2\cdot (CHD-y_{cor})};b_{b}=-2\cdot a_{b}\cdot y_{cor};\\ c_{b}=550-a_{b}\cdot CHD^{2}-b_{b}\cdot CHD;H^{\prime }(CHD)=2\cdot a_{a}\cdot CHD+b_{a}; \end{array}\right.$$

where *HV_sur_* is the hardness on the surface, *HV_cor_* is the hardness in the core, and *y_cor_* and *y_HV_*_,*max*_ denote the depths with hardness equal to *HV_cor_* and the maximum hardness, respectively. In this study, *y_HV_*_,*max*_ is equal to zero. The depth with a hardness of 550 HV is defined as CHD (the case hardening depth). The CHD, *HV_sur_*, and *HV_cor_* are designed as 1.0 mm, 670 HV, and 450 HV, respectively, based on the engineering practice of the crankshaft.

**Figure 8:**
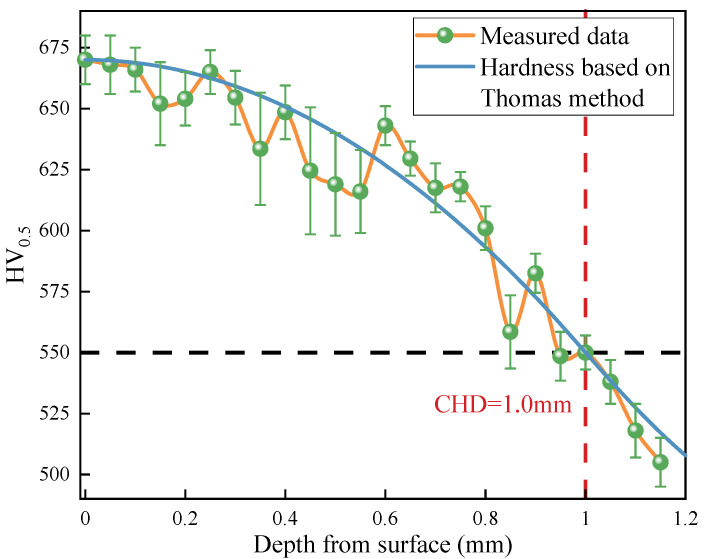
The measured hardness data and the empirical hardness curve.

The crankshaft sample is cut along the cross-section by a wire cutter, inlaid and polished, and then the cross-sectional microhardness is measured by a Vickers hardness tester. A pyramid diamond indenter is selected for the hardness test, with a load of 0.5 kgf and duration of 15 s. Different positions at the same depth were measured at least two times, and the average hardness was obtained. Figure 8 shows the hardness data (the green circle) measured by the hardness tester machine. The red and black dashed lines are used for determine the value of CHD. The empirical hardness gradient curve (the blue solid line) based on the Thomas method is also shown in Figure 8. It can be seen that the empirical curve correlates well with the experimental data.

The tensile strength and yield strength and residual stress distribution will be altered by the variation of martensite, retained austenite, and other structural components during the carburizing-quenching process. A linear relationship existed between Vickers hardness and tensile strength and yield strength, which was given by [27,28].

(5) $$\sigma_{ys}(y)=\frac{HV(y)}{3}\cdot {\left(0.1\right)}^{m-2}$$

(6) $$\sigma_{b}\left(y\right)=\frac{HV\left(y\right)}{3}\left[1-\left(m-2\right)\right]{\left[\frac{12.5\left(m-2\right)}{1-\left(m-2\right)}\right]}^{\left(m-2\right)}$$

where *σ_ys_*(*y*), *HV*(*y*), and *σ_b_*(*y*) are the yield strength, Vickers hardness, and the tensile strength, respectively, and *m* is the Meyer hardness coefficient, which can be taken as 2.19 for high-strength steel materials.

**Figure 9:**
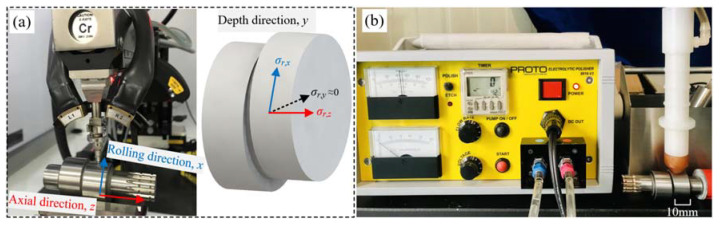
Residual stress measurement: (**a**) X-ray diffractometer; (**b**) Electrolytic polisher.

The generally recognized X-ray diffraction test with electrolytic polisher and empirical formula can be used to determine the residual stress at the contact surface or near-surface areas of the crankshaft. In the present work, the electropolishing method (as shown in Figure 9b) is employed to remove the material layer by layer, and the corresponding residual stress distribution is examined through a Proto iXRD system with Cr-Kα radiation (as shown in Figure 9a). The magnitudes of the residual stress along the *x* and *z* axis (*σ_r,x_* and *σ_r,z_*) are found to be equivalent at the same depth, thus the residual stress is expressed as symbol *σ_r_* in the subsequent section. The residual stress along the depth direction, *σ_r,y_*, is always negligible compared with the other two components, and is thus ignored in this study.

Empirical methods have also been provided to characterize the linear relationship between the hardness curve and the residual stress distribution [29,30]. Hertter’s empirical formula employed in this study is given by [30]:

(7) $$\sigma_{r}(y)=\left\{ \begin{array}{ll} -1.25\cdot (HV(y)-HV_{core}) & \left(HV(y)-HV_{core}\leq 300\right)\\ 0.2857\cdot (HV(y)-HV_{core})-460 & \left(HV(y)-HV_{core}>300\right) \end{array}\right.$$

**Figure 10:**
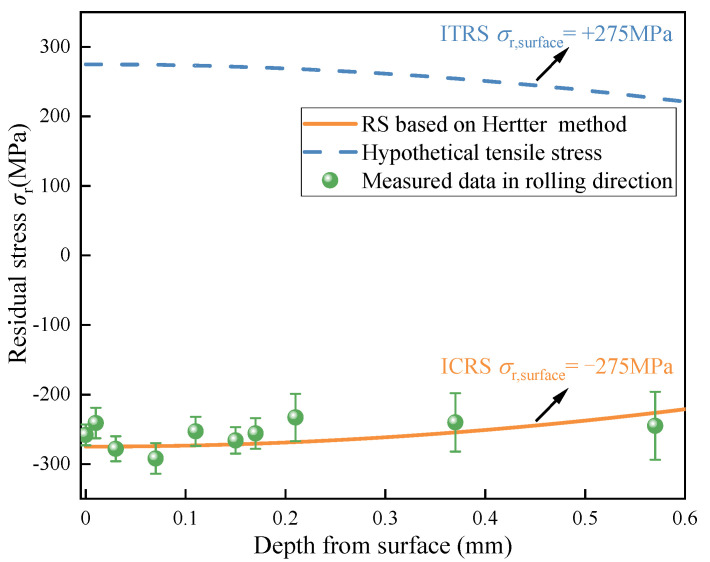
The measured residual stress data and the empirical stress curve.

Figure 10 shows the residual stress data measured by X-ray diffraction (the green circle), and the empirical curve (the yellow solid line) is obtained by the Hertter’s method. It can be observed that the measured residual stresses are, in general, consistent with those fitted by the empirical formula. Therefore, the empirical residual stress curve is employed in the numerical model.

Moreover, the influence of tensile residual stress caused by improper carburizing or grinding burns during the machining process on fatigue damage will also be analyzed in this study [31]. Ultrasonic vibration assisted grinding (UAG) has many advantages, such as reducing grinding force and improving surface quality [32]. A hypothetic tensile residual stress curve (the blue dashed line) with the same amplitude as compressive residual compressive stress is depicted in Figure 10.

**Figure 11:**
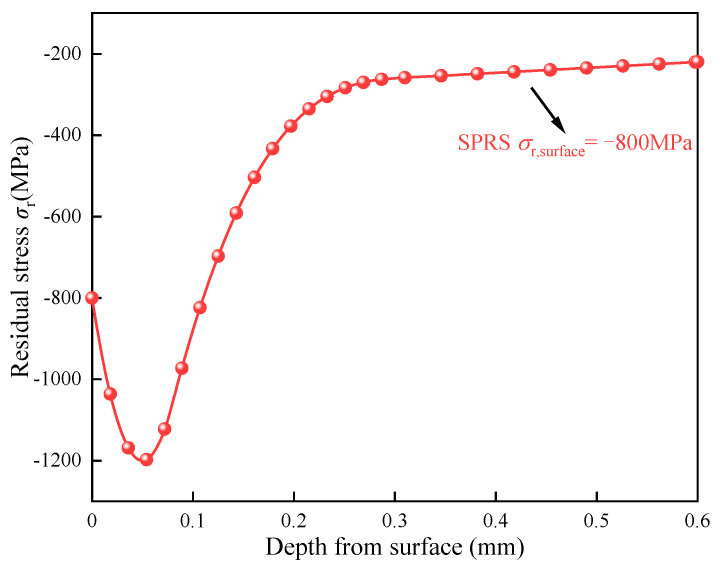
The residual stress distributions induced by shot peening.

The residual stress generated by the plastic deformation near the surface is also caused by surface strengthening processes such as shot peening and ultrasonic rolling. Shot peening produces spoon-shaped residual compressive stress distribution, which is conducive to improving the fatigue performance [33]. Zhao at al. [34] proposed a model for calculating the residual stress distribution after shot peening. The maximum error between the measured and simulated residual stress was 15.8%, which verified the accuracy of the method. A residual stress curve distributed along the depth is designed based on the residual stress data of shot peening in [34], as shown in Figure 11. The maximum plastic deformation near the surface layer leads to the maximum residual compressive stress. The maximum residual stress depth is 50 μm. The compressive stress decreases gradually with the increase of depth and then tends to be stable.

### 3.3. FEM-Based Elasto-Plastic Contact Analysis

**Figure 12:**
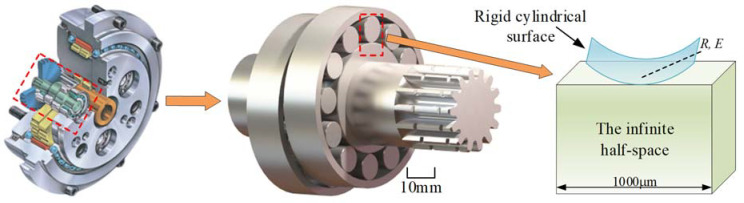
The contact model of the crankshaft and the cylindrical roller bearing.

The rolling contact between the eccentric cylindrical surface of the crankshaft and the cylindrical roller bearing can be simplified as rigid cylindrical surface and a semi-infinite space according to the contact mechanics theory [35]. The diagram of the contact model of the crankshaft and the cylindrical roller is depicted in Figure 12.

**Figure 13:**
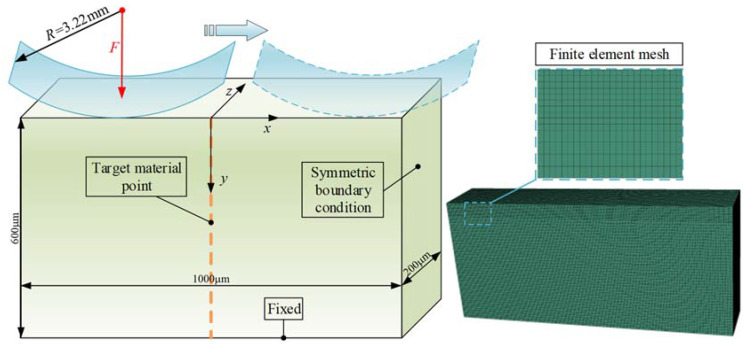
The numerical elasto-plastic contact model.

In order to better reflect the three-dimensional stress–strain field response, a three-dimensional elasto-plastic finite element contact model is established in the commercial finite element software ABAQUS (Figure 13). The equivalent curvature radius of the rigid cylindrical surface is *R* = 3.22 mm. The normal load, *F*, applied on the rigid cylindrical surface, is determined by the output torque of the RV reducer during numerical simulation. The bottom nodes of the mesh model are fully fixed, and symmetric boundary conditions are set around the mesh model. That is, the nodes on the left and right sides of the mesh model are constrained in x and y directions. A static implicit solver is selected for simulation calculation. The computational domain is determined as −500 μm ≤ *x* ≤ 500 μm, 0 μm ≤ *y* ≤ 600 μm, and 0 μm ≤ *z* ≤ 200 μm. The rolling direction, *x*, is long enough to minimize the influence of the boundary on the stress calculation in this direction.

Considering the efficiency and accuracy of finite element calculation, the grid size is gradually expanded from the contact surface to the bottom. The grid size in the *x* and *z* directions is 5 μm. The grid size of the near surface layer in the *y* direction is 5 μm, and the grid size is set to 10 μm as the depth increases. The C3D8R element is selected because it can well withstand distortion, and its stress–strain calculation is also more accurate. The tangential contact property is defined as frictional contact with a friction coefficient of 0.05. The friction coefficient of 0.05, which was also used in [36,37], representing excellent lubrication in rolling contact, is employed in this study. The rigid cylindrical surface rolls from *x* = −400 μm to *x* = 400 μm, and multiple analysis steps are set to realize the reciprocating circular motion of the cylindrical surface. The material points lying on the yellow dashed line (*x* = 0 and *y* ∈ [0, 600] μm) will be analyzed in the present study because the material at the same depth experiences the same cyclical stress.

**Figure 14:**
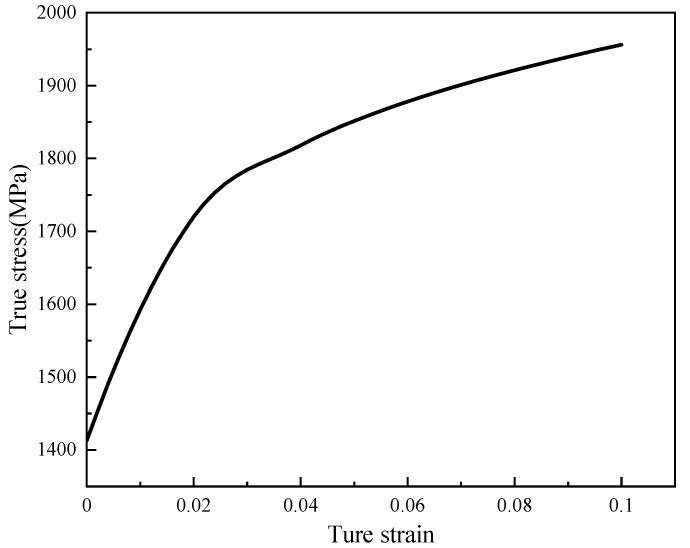
The true stress-plastic strain curve.

Ultra-high stress exists in the kinematic pair (crankshaft and the cylindrical roller bearing) during extreme operation conditions, which inevitably produces plastic deformation. To accurately characterize the elastoplastic response, the varying yield stress with depth is incorporated into the FEM model, according to the linear positive correlation between the hardness and yield strength (Equation (5)). The efficiency for assigning material properties can be improved through the secondary development by Python. Therefore, the isotropic hardening model is applied according to the uniaxial tensile experiment, and the true stress-plastic strain fitting curve is shown in Figure 14. The uniaxial tensile test of material 20CrNi2Mo specimens was completed to investigate the effect of quenching temperature on the properties of strength and toughness [38]. Moreover, in [39], quasi-static tensile/compressive experiments were carried out to obtain the mechanical properties of high strength steel. These experimental results play a great role in this study. Because the yield strength of the bearing rolling element (1617 MPa) is much higher than that of the crankshaft (1413 MPa), contact fatigue failure is more likely to occur on the crankshaft. Therefore, the following mainly analyzes the contact fatigue characteristics of the crankshaft.

**Figure 15:**
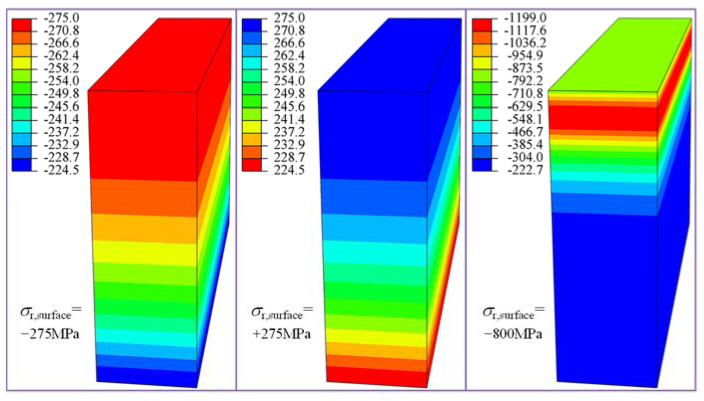
The actual residual stress after equilibrium (without external load).

The initial residual stress field is defined before starting the response history. The hexahedral element has six stress components (*σ*_11_, *σ*_22_, *σ*_33_, *σ*_12,_ *σ*_13,_ *σ*_23_). The shear stress and the stress in the depth direction are negligible according to the above residual stress measurement. Therefore, normal stress components *σ*_11_ and *σ*_33_, confirmed at residual stress *σ_r_* (Figure 10 and Figure 11), are applied to the element integration points of the model (Figure 13). The repetitive work of initial residual stress application can be reduced through the secondary development of Python. After the initial stress field is applied in the finite element model, the equivalent node load formed by the stress field need to be in equilibrium with the specified boundary conditions. Therefore, a static analysis step without external load is set to obtain the actual residual stress field. The actual residual stress obtained by adding different types of residual stress curves (Figure 10 and Figure 11) is shown in Figure 15. The variation of initial residual stress after equilibrium is negligible, which verifies that the method of applying the initial residual stress field to this model is reasonable.

### 3.4. Contact Fatigue Life Assessment Model

The stress in the contact region varies non-proportionally in the loading cycle due to the multiaxial characteristics of the stress state. Thus, it is necessary to apply a preferable multiaxial fatigue criterion to capture stress–strain response and then estimate the fatigue life of the crankshaft in such a complicated time-varying stress state. The FS multiaxial fatigue criterion dominated by shear-type fatigue failure is employed in this work. The normal stress and shear strain with maximum amplitudes are used as fatigue damage parameters. The fatigue life and fatigue damage (*FD*) in this criterion are respectively expressed as [40,41]:

(8) $$\frac{\Delta \gamma_{\max}}{2}(1+k\frac{\sigma_{n,}{}_{\max}}{\sigma_{ys}})=\frac{{\tau^{\prime }}_{f}}{G}{\left(2N_{f}\right)}^{b}+{\gamma^{\prime }}_{f}{\left(2N_{f}\right)}^{c}$$

(9) $$FD_{FS}=\frac{\Delta \gamma_{\max}}{2}[1+k\frac{\sigma_{n,\max}}{\sigma_{ys}}]$$

where Δ*γ*_max_/2 = *γ*_a_ denotes the maximum shear strain amplitude, *σ_n_*_,max_ denotes the maximum normal stress perpendicular to the critical plane, which is assumed to be the plane experiencing maximum shear strain amplitude; *b* and *c* denote the shear fatigue strength and the shear fatigue ductility indexes, set as −0.087 and −0.58 [42]; *G* and *N_f_* are the shear elastic modulus and the crack initiation life, respectively; *k* is the material constant (set as 1 in this study) [43]; and *τ′_f_* and *γ′_f_* denote the shear fatigue strength and shear fatigue ductility coefficients, which are defined as follows [44]:

(10) $${\tau^{\prime }}_{f}={\sigma^{\prime }}_{f}/\sqrt{3},{\gamma^{\prime }}_{f}=\sqrt{3}{\varepsilon^{\prime }}_{f}$$

where *σ′_f_* and *ε′_f_* denote the axial fatigue strength and axial fatigue ductility coefficients, which are calculated by Baumel and Seeger’s method [42]:

(11) $${\sigma^{\prime }}_{f}=1.5\sigma_{b},{\varepsilon^{\prime }}_{f}=0.59\psi$$

(12) $$\psi =\left\{ \begin{array}{ll} 1.0 & (\sigma_{b}/E<0.003)\\ 1.375-125(\sigma_{b}/E) & (\sigma_{b}/E>0.003) \end{array}\right.$$

where *E* is the Young’s modulus and *σ_b_* is the tensile strength.

When analyzing the fatigue data, including combined axial-torsion load paths, it is found that if the maximum normal stress is normalized by the shear stress range, the prediction accuracy of fatigue life can be improved. That is, replacing yield stress, *σ_ys_*, with the shear stress range, GΔ*γ*, the modified fatigue damage (*FD*_mod_) is calculated as follows [45]:

(13) $$FD_{\mathrm{mod}}=\frac{\Delta \gamma_{\max}}{2}(1+k\frac{\sigma_{n,\max}}{G\Delta \gamma })=\frac{{\tau^{\prime }}_{f}}{G}{\left(2N_{f}\right)}^{b}+{\gamma^{\prime }}_{f}{\left(2N_{f}\right)}^{c}$$

The multi-axial stress–strain histories are calculated by using the model outlined in Section 3.3. The stress tensor time history and the strain tensor time history at each element is expressed as *σ*(*t*) and *ε*(*t*), respectively.

(14) $$\sigma (t)=\left[\begin{array}{ccc} \sigma_{xx}(t) & \tau_{xy}(t) & \tau_{xz}(t)\\ \tau_{xy}(t) & \sigma_{yy}(t) & \tau_{yz}(t)\\ \tau_{xz}(t) & \tau_{yz}(t) & \sigma_{zz}(t) \end{array}\right],\varepsilon (t)=\left[\begin{array}{ccc} \varepsilon_{xx}(t) & \frac{1}{2}\gamma_{xy}(t) & \frac{1}{2}\gamma_{xz}(t)\\ \frac{1}{2}\gamma_{xy}(t) & \varepsilon_{yy}(t) & \frac{1}{2}\gamma_{yz}(t)\\ \frac{1}{2}\gamma_{xz}(t) & \frac{1}{2}\gamma_{yz}(t) & \varepsilon_{zz}(t) \end{array}\right]$$

**Figure 16:**
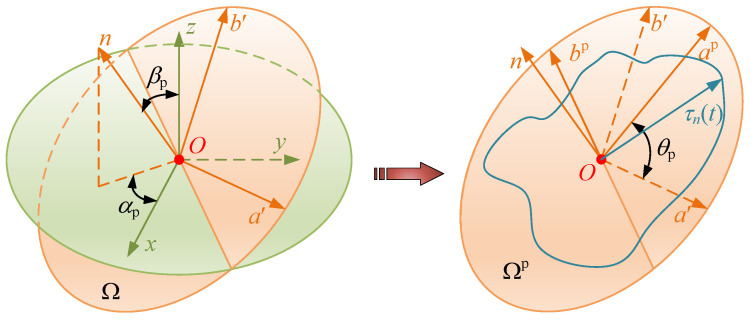
Definition of the Euler transformations and critical plane search method.

Once the multi-axial stress–strain histories are obtained, Euler angle-based axis transformation is employed to search for the critical planes that cover all the possible directions in the 3D space [46], as shown in Figure 16. The transformation of stress vector and strain vector is similar, so the former will be explained in detail below. Considering each element integration point, ***O*** is in the center of the absolute reference frame, **{*O*; *x*, *y*, *z*}**. The orientation of a material plane, **Ω**, with unit normal vector, ***n*** (*n_x_*, *n_y_*, *n_z_*), can be located by using spherical coordinate parameters *α*_p_ and *β*_p_. Parameter *α* is the angle between the projection of unit normal vector ***n*** on plane *x*–*y* and axis *x*, while *β* is the angle between unit vector ***n*** and axis *z* [47].

The transformation coordinate system **{*O*; *a*′, *b*′, *n*}** is defined by searching across two angular ranges (*α*_p_∈ [0, 2π], *β*_p_∈ [0, π]), as shown in Figure 16. Axis *n* is parallel to unit vector ***n***, whereas axes *a***′** and *b***′** are parallel to material plane **Ω**, and the corresponding unit vectors are ***a*** and ***b***, respectively.

(15) $$n=\left[\begin{array}{c} n_{x}\\ n_{y}\\ n_{z} \end{array}\right]=\left[\begin{array}{c} \sin\beta_{p}\cdot \cos\alpha_{p}\\ \sin\beta_{p}\cdot \sin\alpha_{p}\\ \cos\beta_{p} \end{array}\right],\;a=\left[\begin{array}{c} a_{x}\\ a_{y}\\ a_{z} \end{array}\right]=\left[\begin{array}{c} -\sin\alpha_{p}\\ \cos\alpha_{p}\\ 0 \end{array}\right],\;b=\left[\begin{array}{c} b_{x}\\ b_{y}\\ b_{z} \end{array}\right]=\left[\begin{array}{c} -\cos\beta_{p}\cdot \cos\alpha_{p}\\ -\cos\beta_{p}\cdot \sin\alpha_{p}\\ \sin\beta_{p} \end{array}\right]$$

In order to easily calculate the normal stress, *σ_n_*(*t*), and shear stress, *τ_n_*(*t*), associated with the material plane, **Ω**, the total stress vector, ***t***(*t*), depending on the stress tensor, *σ*(*t*), needs to be calculated as:

(16) $$t(t)=\left[\begin{array}{c} t_{x}\left(t\right)\\ t_{y}\left(t\right)\\ t_{z}\left(t\right) \end{array}\right]=\sigma (t)\cdot n=\left[\begin{array}{ccc} \sigma_{xx}(t) & \tau_{xy}(t) & \tau_{xz}(t)\\ \tau_{xy}(t) & \sigma_{yy}(t) & \tau_{yz}(t)\\ \tau_{xz}(t) & \tau_{yz}(t) & \sigma_{zz}(t) \end{array}\right]\left[\begin{array}{c} n_{x}\\ n_{y}\\ n_{z} \end{array}\right]$$

The normal stress and component of shear stress on two axes can be obtained from the above formula, as follows:

(17) $$\{O;a^{\prime },b^{\prime },n\}\Rightarrow \{\begin{array}{l} \sigma_{n}(t)=t_{x}(t)\cdot n_{x}+t_{y}(t)\cdot n_{y}+t_{z}(t)\cdot n_{z}\\ \tau_{\mathrm{na}}(t)=t_{x}(t)\cdot a_{x}+t_{y}(t)\cdot a_{y}+t_{z}(t)\cdot a_{z}\\ \tau_{\mathrm{nb}}(t)=t_{x}(t)\cdot b_{x}+t_{y}(t)\cdot b_{y}+t_{z}(t)\cdot b_{z} \end{array}$$

The shear stress, *τ_n_*(*t*), is obtained as follows:

(18) $$\tau_{n}(t)=\sqrt{\tau_{na}^{2}(t)+\tau_{nb}^{2}(t)}$$

During the load cycle, normal stress, *σ_n_*(*t*), varies its magnitude, but the direction remains parallel to vector ***n***. Therefore, its maximum value can simply be calculated as follows:

(19) $$\sigma_{n,\max}=\max[\sigma_{n}(t)]$$

Meanwhile, shear stress, *τ_n_*(*t*), varies its magnitude and direction with time, which makes the solution of the shear stress amplitude more complicated. This coordinate system, **{*O*; *a*′, *b*′, *n*}**, is then rotated about axis *n* until the amplitude of the shear stress component along axis *a*^p^ is maximized. The new coordinate system is recorded as **{*O*; *a*^p^, *b*^p^, *n*}**, and **Ω_p_** is used to represent the critical plane, as shown in Figure 16, assuming that the rotation angle is *θ_p_* (*θ_p_* ∈ [0, π]) and the transformation matrix is **T**(*θ_p_*), defined by:

(20) $$T(\theta_{p})=\left[\begin{array}{ccc} 1 & 0 & 0\\ 0 & \cos\theta_{p} & \sin\theta_{p}\\ 0 & -\sin\theta_{p} & \cos\theta_{p} \end{array}\right]$$

The stress tensors around this coordinate system are given as:

(21) $$\{O;a^{p},b^{p},n\}\Rightarrow \{\begin{array}{l} \sigma_{n}^{P}(t)=T(\theta_{p})\cdot \sigma_{n}(t)\\ \tau_{na}^{P}(t)=T(\theta_{p})\cdot \tau_{\mathrm{na}}(t)\\ \tau_{nb}^{P}(t)=T(\theta_{p})\cdot \tau_{\mathrm{nb}}(t) \end{array}$$

That is, the *θ_p_* that maximizes the in-plane shear stress amplitude along axes *a*^p^ is searched for, and at this moment the amplitude of $\tau_{nb}^{p}(t)$ is the smallest [48]. Thus, the stress vector satisfying the following relationship can be obtained:

(22) $$\tau_{n,a}=\frac{1}{2}[\max\tau_{n}(t)-\min\tau_{n}(t)]$$

**Figure 17:**
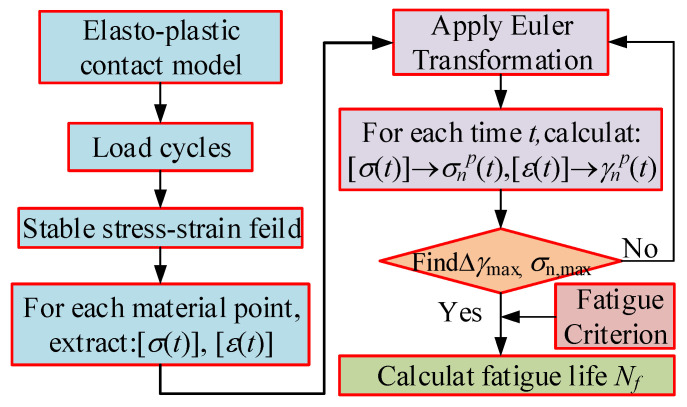
Computational methodology of predicting fatigue life.

The overall computational methodology of estimating the fatigue life demonstrated above is summarized in the flow chart of Figure 17. Firstly, the stable stress–strain field is calculated by the elasto-plastic contact model, and the stress–strain components of the target material pointing along the depth is extracted through the secondary development of Python. Then, the stress, strain (*σ_n_*_,max_ and Δ*γ*_max_), and *FD*_mod_ are calculated. Finally, the fatigue initiation life is evaluated via the Newton–Raphson method.

## 4. Results and Discussion

### 4.1. Effect of Friction Coefficient and Normal Stress on Fatigue Damage

**Figure 18:**
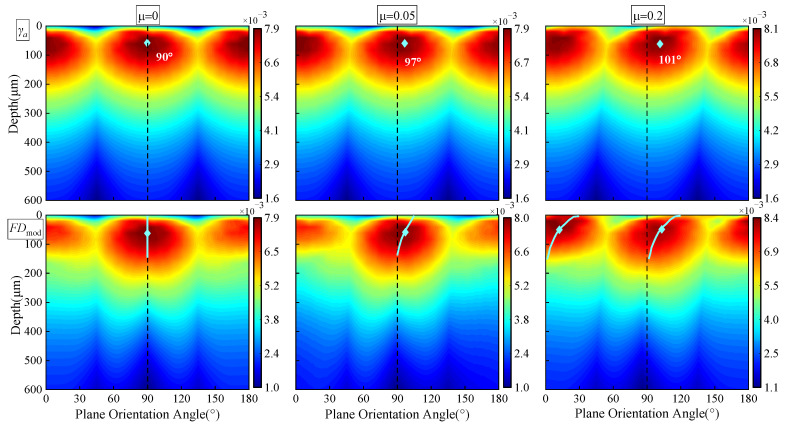
Variation of *γ*_a_ and *FD*_mod_ under different lubrication conditions (*T_out_* = 800 N·m, *P_h_* = 1.56 GPa).

The friction coefficients of 0, 0.05, and 0.2 correspond to ideal smooth contact without friction, the reasonable friction coefficient for electrohydrodynamic lubricated bearing contact, and the state where the excellent lubrication environment of the reducer is destroyed. These three friction coefficients are selected firstly to investigate the effect of lubrication on the shear strain, *γ*_a_, and the modified fatigue damage, *FD*_mod_, on the material plane (*θ_p_* ∈ [0, π]) with the initial compressive residual stress (ICRS) case under the framework of modified FS criterion. The output torque is set as the rated output torque of 800 N·m and the Maximum contact stress *P_h_* = 1.56 GPa. As can be seen from Figure 18, the contour of shear strain amplitude, *γ*_a_, is symmetrical, and *γ*_a,max_ lies at the plane orientation angles of 90° and 0° (180°) when the friction coefficient, *μ*, is equal to 0. The distribution of *γ*_a_ and *FD*_mod_ over *θ_p_* is asymmetric when *μ* ≠ 0. Moreover, the worse lubrication, with a friction coefficient of 0.2, leads to a much more obvious asymmetry of the *FD*_mod_ contour. According to the definition of the critical plane, the plane orientations are along *θ_p_ =* 97° and *θ_p_ =* 101° when the friction coefficients are 0.05 and 0.2. The location of the maximum damage gradually moves to the contact surface, and the fatigue damage increases as the friction coefficient increases; similar results are also found in Ref [49].

**Figure 19:**
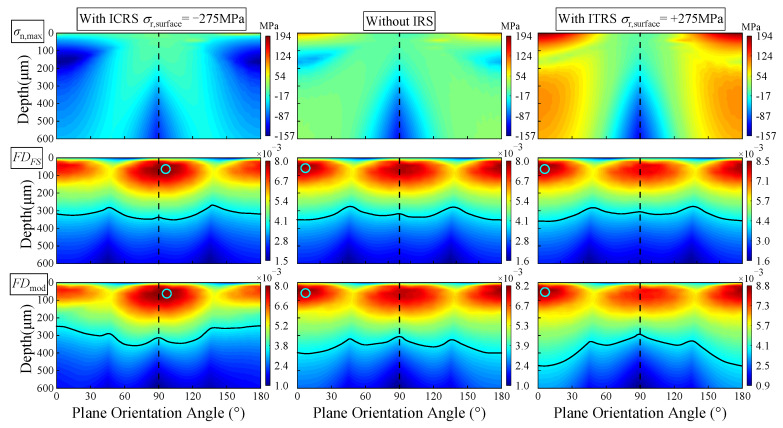
Effect of initial residual stress on *σ_n_*_,max_ and the fatigue damage (*T_out_* = 800 N·m).

Three residual stress conditions corresponding to the residual stress curves shown in Figure 10, such as initial compressive residual stress (ICRS), without initial residual stress (IRS), and initial tensile residual stress (ITRS), are chosen to explore the influence of initial residual stress on the fatigue damage (Figure 19). The output torque and friction coefficient are set as 800 N·m and 0.05, respectively. It can be observed from Figure 19 that the distributions of the maximum normal stress under three initial residual stress states are completely different at the subsurface, and the initial residual stress states appreciably influences the maximum normal stress, *σ*_max_, near the *θ*_p_ = 0° (180°) plane. Compared with the without the IRS case, ICRS reduces *σ*_max_ by 48.4% while ITRS increases the value of *σ*_max_ by 99.8%.

The variations of FS damage (*FD*_FS_) and modified FS damage (*FD*_mod_) are also plotted versus plane orientation in Figure 19, which can provide additional insight into the damage mechanisms. Figure 19 shows that the variations of damage value against different plane angles under the modified FS criterion is more obvious than that under the FS criterion. The maximum fatigue damage, *FD*_mod,max_, occurs at the *θ_p_* = 97° plane with the ICRS case, while the maximum damage, *FD*_mod,max_, shifts from *θ_p_* = 97° to *θ_p_* = 5° as the initial residual stress changes from compressive stress to tensile stress. This might be attributed to the fact that the increase of maximum normal stress leads to the increase of the damage near the *θ_p_* = 0° (180°) plane. Therefore, the initial residual stress affects the position of *FD*_mod,max_ by influencing the distribution of the maximum normal stress under the FS criterion; similar results are also found in [50]. Moreover, the tensile normal stress with the ITRS case results in higher damage predictions near the 0° (180°) plane for modified FS damage. Tensile stress help to accelerate shear crack growth, while compressive normal stress with the ICRS case may serve to increase friction between the cracks and reduce the crack driving force. The above also demonstrates that the ratio of maximum normal stress to shear stress in modified FS damage (*FD*_mod_) can better consider the interaction effect between the two stress [45]. Therefore, the ICRS would lead to a decrease in fatigue damage and increase the crack initiation life, *N_f_*.

### 4.2. Effect of Initial Residual Stress on Plastic Strain

**Figure 20:**
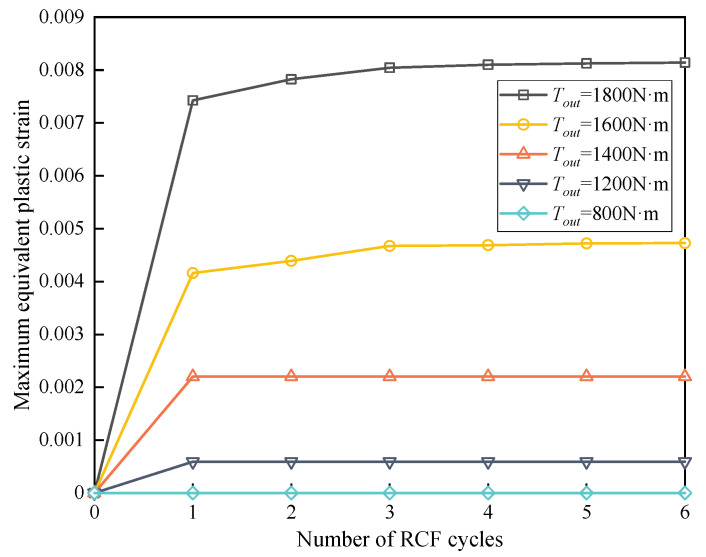
Effect of output torque on the maximum equivalent plastic strain (Max PEEQ).

The equivalent plastic strain is chosen as the evaluation index for the analysis of contact fatigue damage. Figure 20 illustrates the variation of the maximum equivalent plastic strain (Max PEEQ) corresponding to different output torques in the RCF cycles. The Max PEEQ is close to 0 when the output torque is small, indicating that a pure elastic response exists under this circumstance. It is evident that the Max PEEQ increases remarkably with the increase of output torque. It can be concluded that heavy load conditions would aggravate the rate of plastic strain accumulation, resulting in the redistribution of stress.

**Figure 21:**
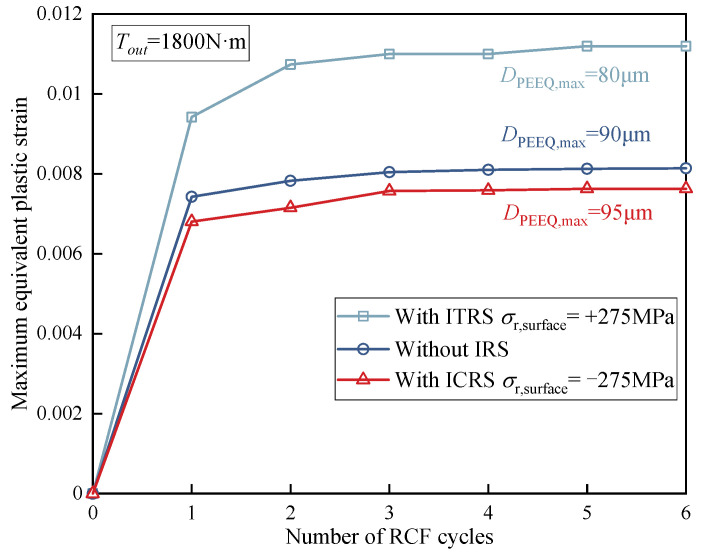
The effect of initial residual stresses on the maximum equivalent plastic strain (Max PEEQ) in RCF cycle (*T_out_* = 1800 N·m, *P_h_* = 2.34 GPa).

Figure 21 demonstrates the variation of the maximum equivalent plastic strain (Max PEEQ) corresponding to different residual stress cases in the RCF cycles when the output torque is set as *T_out_* = 1800 N·m and the maximum contact stress *P_h_* = 2.34 GPa. Compared with the case without IRS, ICRS reduces PEEQ by 6.3%, while ITRS increases PEEQ by 37.5%. The increase rate of PEEQ for ITRS is obviously greater than the decrease rate of PEEQ for ICRS. RCF behaviors such as pitting and spalling more likely appear near the surface owing to the position of Max PEEQ near to the contact surface with ITRS. Meanwhile, the presence of ICRS makes the position where the maximum equivalent plastic strain appear deeper. It is also found in [14] that the compressive residual stresses make the crack initiation depth deeper. Because the crack initiation depth determines the crack propagation length required to reach the surface, the ICRS enhanced the fatigue life by prolonging the number of cycles of cracks reaching the surface.

**Figure 22:**
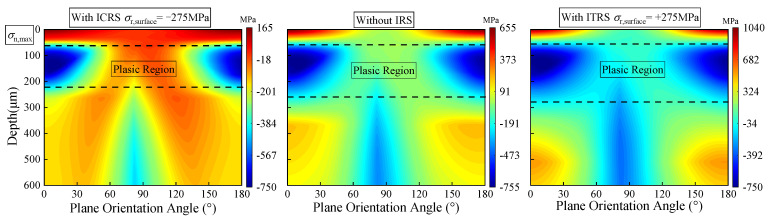
The effect of residual stress on the plastic region scope (*T_out_* = 2000 N·m, *P_h_* = 2.47 GPa).

The initial residual stress also significantly affects the scope of the plastic region. Figure 22 depicts the variation of the maximum normal stress corresponding to different residual stress cases when the output torque is set as *T_out_* = 2000 N·m (*P_h_* = 2.47 GPa). It can be clearly seen from the figure that the plastic region forms at a certain depth of material rather than initiates on the surface. Under the same loading condition, the presence of ICRS shrinks the plastic region, while ITRS expands the plastic region compared with that without IRS. Almost the same maximum normal stress in the plastic region (around −750 MPa) are found for all the three scenarios. However, the distribution and magnitude of the maximum normal stress at a depth of 40 μm beneath the surface are totally different. The maximum normal stress in this region for ITRS is the largest, while the increase of the maximum normal stress is inhibited by the ICRS.

### 4.3. Effect of Initial Residual Stress on Fatigue Life

**Figure 23:**
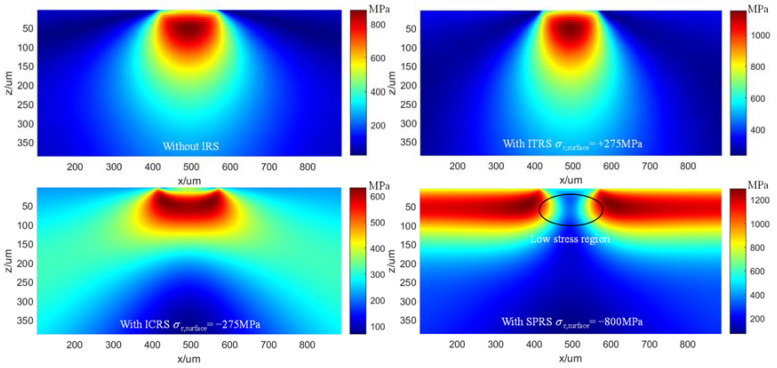
The effect of initial residual stress on the von Mises stress field (*P_h_* = 1.56 GPa).

The von Mises stress field and fatigue life corresponding to different initial residual stress is further investigated in this section. Figure 23 shows the von Mises stress field at the contact center of the x–z section when different initial residual stress states are applied. It is worth noting that the SPRS represents the residual stress introduced by shot peening (as shown in Figure 11). The maximum von Mises stress under the contact center in IRS state is 615.9 MPa, which is 30.4% lower than that without IRS, while the von Mises stress increase amplitude in ITRS state is 30.7% higher than that without IRS. What is more noteworthy is that there is an obvious low stress region under the contact center with the SPRS state. The maximum von Mises stress is an effective criterion to completely avoid the occurrence of plastic deformation. The significant reduction of the maximum von Mises stress means that the occurrence of plastic deformation can be suppressed or delayed [17]. The application of appropriate initial compressive residual stress can significantly reduce the plastic deformation and thus improve the contact fatigue performance.

**Figure 24:**
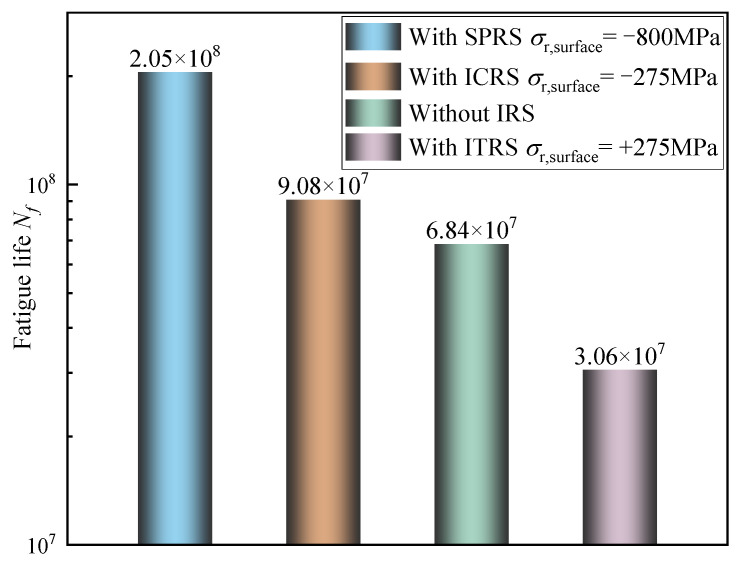
Variation of contact fatigue life with different initial residual stress states (*P_h_* = 1.56 GPa).

Figure 24 illustrates the minimum fatigue life, namely the fatigue limit on the basis of the modified FS criterion when the output torque is set as 800 N·m. Under the rated load condition, the minimum contact fatigue life with the ICRS case is 9.08 × 10^7^, which is 32.7% larger than that without IRS, while the fatigue life with ITRS is 55.3% lower than that without IRS. It is apparent that the fatigue life after applying residual stress introduced by shot peening is increased by 125.7% compared with the state with ICRS. Therefore, the fatigue life can be considerably improved by designing an appropriate shot peening process and optimizing residual stress distribution.

Based on the modified Fatemi–Socie (FS) multiaxial fatigue criterion, the fatigue life forecasting has considerably improved by taken hardness gradients and initial residual stress into account especially under multiaxial loading conditions. Under the rated output torque of 800 N·m, the experimental fatigue life of the RV reducer is 2150 h (7.55 × 10^7^). The error between the predicted fatigue life (9.08 × 10^7^)and the experimental value is 20.3%. Considering the complex environmental conditions and the fatigue crack growth path in the actual work process of the RV reducer, the calculation results under this error are acceptable. However, there are still many shortcomings in the proposed method, such as the wear evolution behavior of morphology during service, contact pressure distribution under lubrication conditions, cumulative calculation of fatigue damage, etc. Fatigue life assessment would be more challenging considering the coupling effect of these factors.

## 5. Conclusions

In this work, focusing on fatigue life assessment of an RV reducer crankshaft, an innovative FEM-based three-dimensional elasto-plastic contact model is proposed. The RCF characteristics of the crankshaft are investigated by applying the modified Fatemi–Socie (FS) multiaxial fatigue criterion. Some distinct features can be highlighted, as follows:

(1) The location of the maximum shear strain depends on the friction coefficient. When the friction coefficient is low, the position of the maximum shear strain is still on the subsurface. Meanwhile, the fatigue initial location moves from the subsurface to the surface, and the fatigue damage increases as the friction coefficient *μ* increases.
(2) The initial residual stress plays an influential role in fatigue damage and crack initiation depth by altering the distribution of the maximum normal stress, *σ_n_*_,max_, near the contact surface. The compressive residual stress can reduce *σ_n_*_,max_ by 48.4% compared with that without residual stress. Therefore, the ratio of maximum normal stress to shear stress in the modified FS fatigue criterion can better consider the interaction effect between the residual stress and the shear stress, which significantly improves the prediction accuracy of the contact fatigue life model.
(3) The ICRS makes the plastic region shrink and improves the contact fatigue performance by delaying the time of cracks propagating to the surface. Under the rated load condition, the minimum contact fatigue life with ICRS is 9.03 × 10^7^, which is 29.6% larger than that without IRS, while the minimum contact fatigue life with SPRS is 125.7% larger than that with ICRS. Residual stress distribution introduced by shot peening significantly enhances the fatigue life of the crankshaft.
(4) Moreover, the fatigue life could be maximized by designing appropriate shot peening parameters to obtain optimized residual stress distribution. The experimental verification of the proposed fatigue life assessment method can also be conducted in future study though it is costly and time-consuming.

## Figures and Tables

**Table 1:** Crankshaft-bearing rolling pair parameters.

Parameters	Value	Parameters	Value
Crankshaft rotation speed (r/min)	*n_s_* = 585	Rated output torque (N·m)	*T_out_* = 800
Radius of the rolling element (mm)	*R*_1_ = 4.0	Rolling element material	GCr15
Radius of the eccentric cylindrical surface (mm)	*R*_2_ = 16.6	Crankshaft material	20CrNi2MoA
Length of the rolling element (mm)	*l*_1_ = 12.0	Young’s modulus (GPa)	*E*_1_ = 219, *E*_2_ = 210
Radius of needle tooth distribution circle (mm)	*R_z_* = 82	Poisson ratio	*v*_1_ = 0.3, *v*_2_ = 0.275
Radius of crankshaft distribution circle (mm)	*r_o_* = 46.77	Eccentricity (mm)	*e* = 1.5
Number of teeth of needle wheel	*z*_4_ = 39	Number of crankshafts	*n* = 3
Number of teeth of cycloid gear	*z*_5_ = 40	Short amplitude coefficient	*k* = 0.7317

**Table 2:** The composition of 20CrNi2MoA.

Element	C	Mn	Cr	Ni	Si	Mo	Cu	S	P	Fe
Wt.%	0.21	0.63	0.57	1.8	0.33	0.25	0.30	0.015	0.02	Bal.

## Data Availability

Not applicable.
